# Pomalidomide Treatment in Relapsed/Refractory Multiple Myeloma Patients—Real-World Data From Hungary

**DOI:** 10.3389/pore.2022.1610645

**Published:** 2022-10-03

**Authors:** Szilvia Lovas, Nóra Obajed Al-Ali, Gergely Varga, Virág Szita, Hussain Alizadeh, Márk Plander, Péter Rajnics, Árpád Illés, Zsuzsa Szemlaky, Gábor Mikala, László Váróczy

**Affiliations:** ^1^ Department of Hematology, Institute for Medicine, Faculty of Medicine, University of Debrecen, Debrecen, Hungary; ^2^ Department of Internal Medicine and Hematology, Semmelweis University, Budapest, Hungary; ^3^ 1st Department of Internal Medicine, University of Pécs, Pécs, Hungary; ^4^ Department of Hematology, Markusovszky Teaching Hospital, Szombathely, Hungary; ^5^ Department of Hematology, Mór Kaposi Teaching Hospital, Kaposvár, Hungary; ^6^ Department of Hematology and Stem Cell Transplantation, South-Pest Central Hospital, National Institute for Hematology and Infectious Diseases, Budapest, Hungary

**Keywords:** survival, toxicity, multiple myeloma, treatment response, pomalidomide

## Abstract

Pomalidomide is a third generation immunomodulatory drug in the treatment of refractory and relapsed multiple myeloma patients. Our aim was to investigate the efficacy and safety of pomalidomide therapy in a real world setting. Eighty-six Hungarian patients were included, 45 of whom received pomalidomide ± an alkylating agent, while in 38 of them pomalidomide was combined with a proteasome inhibitor. 56 patients (65%) showed any response to the treatment with 18 complete or very good partial remissions and 38 partial remissions. At a median duration of follow-up of 18.6 months, the median progression-free survival (PFS) was 9.03 months, while the median overall survival (OS) was 16.53 months in the whole cohort. Patients with early stage disease (R-ISS 1 and 2) had better survival results than those with stage 3 myeloma (*p* = 0.002). Neither the number of prior treatment lines, nor lenalidomide refractoriness had a significant impact on PFS*.* PFS was found similar between the cohort of patients with impaired renal function and the cohort without kidney involvement. During the study, eight mortal infections and two fatal bleeding complications occurred, however, mild hematologic and gastrointestinal toxicities were identified as the most frequent adverse events. The results of our investigations confirm that pomalidomide is an effective treatment option for relapsed/refractory MM, besides, the safety profile is satisfactory in subjects with both normal and impaired renal function.

## Introduction

Multiple myeloma (MM) is a clonal plasma cell disorder that accounts for ten per cent of all hematological malignancies [1]. The major complications associated with the disease include lytic bone lesions, hypercalcaemia, bone marrow failure and renal impairment [1]. Although the condition is regarded incurable, there has been a remarkable improvement in patients’ survival, due to the novel pharmacological therapies that have been recently introduced [1].

Besides proteasome inhibitors (PI) and monoclonal antibodies, immunomodulatory drugs (IMids) represent the backbone of therapy in all treatment lines [1]. Thalidomide, the first IMid introduced more than 20 years ago, was followed by the new generation representatives lenalidomide and pomalidomide [2]. All IMids have pleiotropic effects that include direct induction of apoptosis in malignant tumor cells, interference with plasma cell and bone marrow stromal cell interactions and the boost of the anti-tumor immune responses [2]. Their primary target is a protein called cereblon (CRBN). The IMids bind the CRL4CRBN E3 ligase, which leads to the ubiquitination of transcription factors Ikaros (IKZF1) and Aiolos (IKZF3) and results in the activation of several anti-proliferative pathways [3].

Pomalidomide is a 3rd generation IMid that was approved in the treatment of relapsing-refractory setting of multiple myeloma by the FDA in 2013 [4]. Its conventional daily dose is 4 mg that can be gradually reduced to 1 mg. Its half life is 7.5–9.5 h. It is excreted both in the kidneys and the liver, however, dose reduction is recommended in patients with kidney failure [4]. The main side effects include myelosuppression, fatigue and diarrhea [4]. Pomalidomide can be administered with dexamethasone or in combination with proteasome inhibitors (bortezomib) and monoclonal antibodies (isatuximab, daratumumab) [5].

The aim of this study was to evaluate the efficacy and safety of pomalidomide-based therapies in a large cohort of Hungarian relapsed/refractory MM patients.

### Patients, Procedures and Methods

We approached all centers in Hungary where pomalidomide was used, and retrospective data were available for collection and analysis. The clinical files of multiple myeloma patients were reviewed with the focus on age, sex, clinical stage, renal function, response to treatment and survival. ISS stages were determined according to the International Myeloma Working Group (IMWG) diagnostic criteria.

FISH testing was performed according to local protocols which vary and are specific to each of the participating center. Even if no agreement exists between the centers regarding the probes used, those for 17p deletion, translocations (11; 14), (4; 14) and (14; 16) and 1q amplification were mutually approved and used. FISH results of unfavourable survival outcome included t(4; 14), t(14; 16), del(17p) and amp(1q21).

Patients received pomalidomide orally on days 1–21 of each 28-day cycle in the dose of 4 mg, combined with dexamethasone 40 mg weekly plus or minus a third agent. Prophylactic anticoagulation was administered in every case unless any contraindications applied. The anticoagulants that were used could be aspirin, low molecular weight heparin or vitamin K antagonists, depending on the patients’ risk factors and comorbidities. A precise comparison of treatment efficacy was guaranteed by defining response criteria (complete response [CR], very good partial response [VGPR], partial response [PR], no response [NR], and progressive disease [PD]) and survival measures (progression-free survival [PFS] and overall survival [OS]) in accordance with published IMWG guidelines. Overall survival (OS) was measured from diagnosis until the event of death from any cause, while progression-free survival (PFS) was measured until the first documented signs of relapse, disease progression indicating further treatment or the event of death. In our survival analysis, survival rates in the cohorts were calculated using the Kaplan-Meier method, while survival estimates were compared using the log-rank test. Differences with probability value of less than 5% were regarded as statistically significant (*p* < 0.05).

## Results

### Patient Characteristics

Six centers were involved in the trial with 86 patients in total, all of whom received pomalidomide-based therapies between July 2018 and December 2021. All medical centers applied balanced patient-level demographic characteristics (Table 1). 55.8% of the subjects were male. Average age at the start of pomalidomide therapy was 62.16 ± 8.7 years (median: 62; range: 42–83). Patients were heavily pretreated, the median number of prior lines was 4 (range: 2–12). All patients had prior bortezomib and lenalidomide treatment and 87% of them were refractory to the latter. Other prior therapies included daratumumab (57%), carfilzomib (39%) and ixazomib (28%), as well as a few patients who had received novel innovative drugs such as isatuximab, venetoclax, selinexor or belantamab-mafodotin. 62.7% of the patients had prior autologous stem cell transplantation. Pre-pomalidomide FISH tests were available for 78 patients, 53 (61.6%) of which showed high risk cytogenetics. The majority had high international staging system (ISS) score, with 24, 29 and 33 patients in the ISS 1, 2 and 3 groups respectively. Markedly impaired renal function (GFR< 30 ml/min) has been reported in 22 cases. 27 patients had extramedullary manifestation of the disease when pomalidomide therapy was initiate. To detect extramedullary disease, mainly CT scan and MRI were used, however, PET-CT was performed in 8 cases. Three of these patients had central nervous system involvement.

**TABLE 1:** Demographic and clinical characteristics of the patient population (ASCT = autologous stem cell transplantation).

Characteristic	
Age at the diagnosis(years)	
Median (range) distribution, n	62 (42–83)
<65	50
65–74	27
75≤	9
Type of measurable disease n (%)	
IgG	47 (54.65)
IgA	20 (23.25)
Light-chain	19 (22.1)
ISS disease staging n	
I	24
II	29
III	33
Number of prior lines of therapy	
median (range)	4 (2–12)
Prior treatments n (%)	
Bortezomib	86(100)
Thalidomide	75 (87.2)
Lenalidomide	85 (98.83)
ASCT	56 (65.11)
Pre-Pomalidomid cytogenetic profile, n (%)	
standard risk cytogenetic abnormality	25/86 (29.07%)
high risk cytogenetic abnormality	53/86 (61.6%)
Unknown	9/86 (10.47%)

### Adverse Events

According to the reported number of adverse events (AEs), pomalidomide treatment was well tolerated. AEs above grade 1–2 ocurred in 33 cases (38%). The most frequent AEs were cytopenias (grade 1–2) and gastrointestinal toxicities (diarrhea).

There were eight fatal infections that included seven cases of pneumonia and related septicaemia and two cases of fatal bleeding. COVID infection occurred in ten patients, two of them resulted in death (Table 2).

**TABLE 2:** Adverse events.

	Grade 1–2	Grade 3	Grade 4	Grade 5
Neutropenia	41	6	1	
Thrombocytopenia	34	8	1	
Anaemia	43	1		
Infection pneumonia	12	2	1	8
		2	1	8
COVID	6	1	1	2
Gastrointestinal	32	1		
Bleeding			1	2
All	162	18	5	10

### Efficacy

**FIGURE 1:**
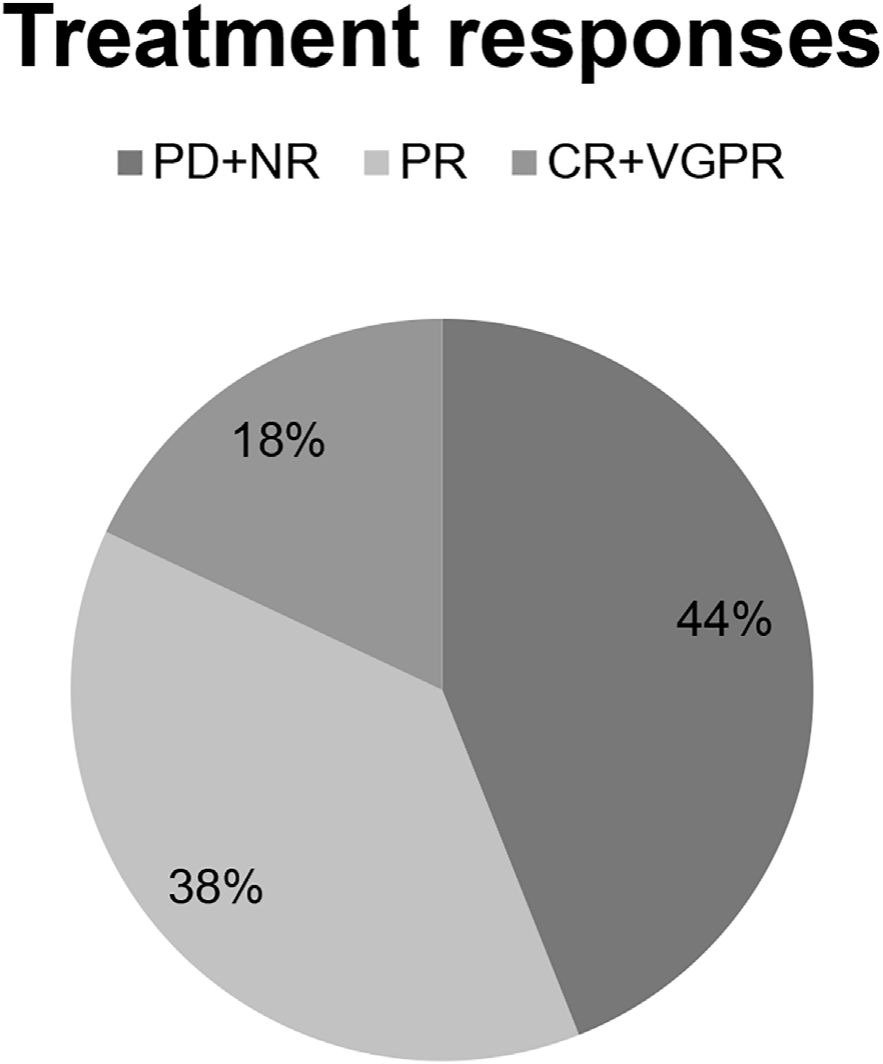
Treatment response rates.

The majority of patients (45 cases, 52.39%) received a combination with only dexamethasone ± an alkylating agent, but 38 of them (44.1%) had a PI (bortezomib, carfilzomib or ixazomib) in addition. In three cases, pomalidomide was combined with other agents (daratumumab, venetoclax or pembrolizumab). Treatment was usually continued until progression, unacceptable toxicity or death. Dose reduction was necessary in 21 cases due to mild or moderate toxicities. The median number of cycles was 7 (1–41). Overall response rate was assessable in all patients, with 18% having complete or very good partial and 38% partial remission. 44% of the patients showed minor or no response to the pomalidomide therapy (Figure 1).

**FIGURE 2:**
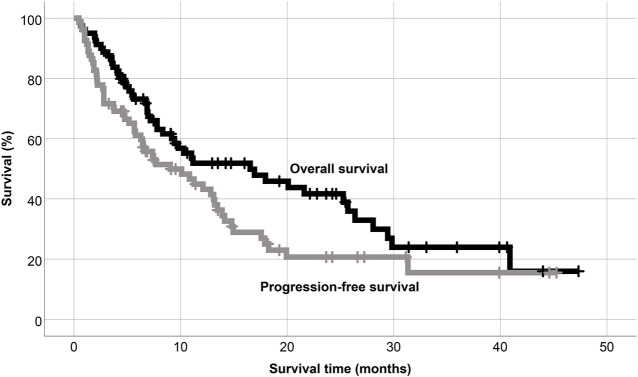
Overall and progression free survival of the whole population.

**FIGURE 3:**
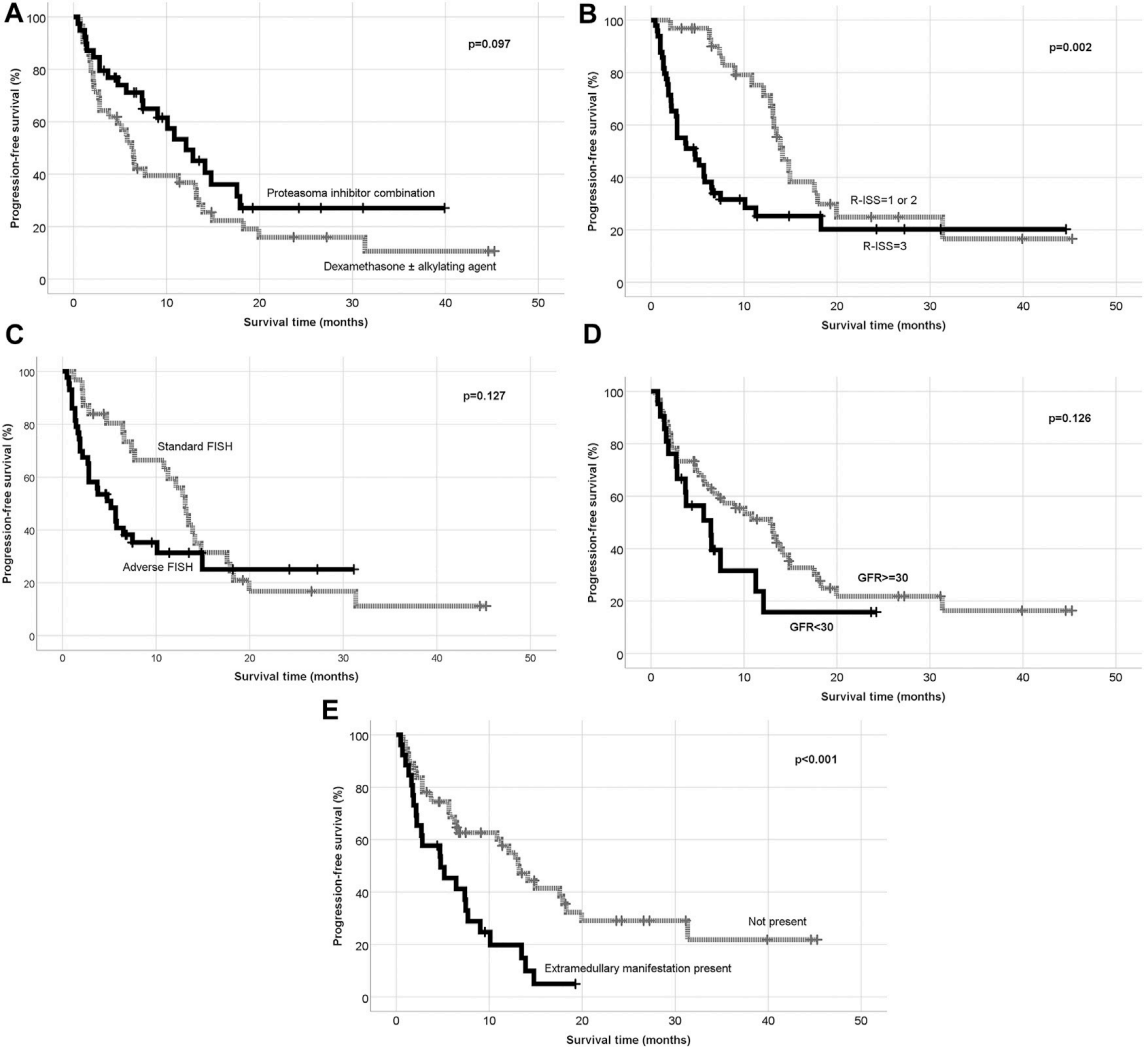
**(A)** Progression free survival according to the treatment combination. **(B)** Progression free survival according to R-ISS. **(C)** Progression free survival according to the FISH results. **(D)** Progression free survival according to the renal function. **(E)** Progression free surivival according to extramedullary manifestation.

The clinical cut-off date was 31 December 2021. At a median follow-up period of 18.6 months (range 1–30), the median PFS was found 9.03 months, while the median OS was measured 16.53 months in the whole cohort (Figure 2). On the basis of our results, that there was a trend towards superior PFS in the PI combination group (Figure 3A). Patients with earlier stage disease (R-ISS 1 and 2) had significantly better survival results than those with R-ISS stage 3 myeloma (Figure 3B). The number of prior therapies did not seem to influence the PFS results and there was no significant difference between the survival rates of lenalidomide-refractory and non-refractory patients either. In terms of the FISH results, patients having adverse genetic alterations showed markedly inferior survival in comparison with standard FISH results (post hoc analysis, *p* = 0.127) (Figure 3C)*.* Notably, patients with severe renal function impairment showed similar PFS results to those without renal dysfunction (Figure 3D). However, extramedullary manifestation of multiple myeloma remained an adverse prognostic marker in patients receiving pomalidomide therapy as well (Figure 3E).

## Discussion

Pomalidomide was approved for the treatment of RR multiple myeloma patients by the FDA in 2013*.* A randomized phase 3 trial (MM03 or NIMBUS) was conducted to assess the efficacy and safety of pomalidomide plus low dose dexamethasone (Pom/Dex) versus high-dose dexamethasone (HDD) in RRMM patients who underwent and failed prior bortezomib and lenalidomide therapies. The results of the investigation revealed that low dose Pom/Dex showed significantly longer median PFS (4.0 months vs. 1.9 months) and median OS (12.7 months vs. 8.1 months) than HDD. These findings were promising, however, the expected survival was still poor in this heavily pretreated population [6]. The phase 3 OPTIMISMM trial targeted myeloma patients who had already received one to three previous lines of therapies, including a lenalidomide-containing regimen for at least two consecutive cycles. 70% of the patients were lenalidomide refractory. Patients were assigned either to pomalidomide – bortezomib – dexamethasone (PVd) triplet therapy or to bortezomib - dexamethasone (Vd) combination. PVd treatment significantly improved PFS in lenalidomide-refractory and non-refractory patients, compared to the results achieved with Vd therapy (12.0 and 22.0 months vs. 5.59 and 9.53 months, respectively. Evaluation of treatment also revealed that patients who had received one previous line of treatment and were refractory to lenalidomide reached a median progression-free survival of 17.84 months with PVd versus 9.49 months with Vd. Improved progression-free survival with pomalidomide, bortezomib, and dexamethasone versus bortezomib and dexamethasone was also observed in several clinically relevant prespecified subgroups, including patients with high-risk cytogenetics (median 8.44 months vs. 5.32 months) and those with previous exposure to proteasome inhibitors (median 10.91 months vs. 6.31 months) [7].

The introduction of monoclonal antibodies in the treatment of RR multiple myeloma opened new possibilities for the creation of novel innovative pomalidomide-containing regimens. In the ICARIA phase 3 trial, eligible participants were patients who had received at least two previous lines of treatment including lenalidomide and a proteasome inhibitor. They were randomly assigned to either the anti-CD38 monoclonal antibody isatuximab plus pomalidomide and dexamethasone or pomalidomide and dexamethasone therapy. At a median follow-up of 11.6 months median progression-free survival was 11.5 months in the isatuximab – pomalidomide – dexamethasone group versus 6.5 months in the pomalidomide–dexamethasone group. The results demonstrated that the progression-free survival benefit with isatuximab was consistent in all prespecified subgroups, including patients with poor prognosis; those refractory to lenalidomide, a proteasome inhibitor, or both lenalidomide and a proteasome inhibitor at the last line previous to study entry [8]. In the APOLLO phase 3 study, daratumumab, a different combination therapy was applied: the patients received anti-CD38 monoclonal antibody which was combined with pomalidomide. Eligibility criteria were defined as the following: the study population only included patients who had relapsed or refractory multiple myeloma with measurable disease, had received at least one previous line of therapy with both lenalidomide and a proteasome inhibitor, had a partial response or better to one or more previous lines of anti-myeloma therapy, and were refractory to lenalidomide if they had received only one previous line of treatment. Patients were randomly selected and divided into two groups. Subjects in these groups were assigned (1:1) to receive daratumumab plus pomalidomide and dexamethasone or pomalidomide and dexamethasone alone, stratified by number of lines of previous therapy (1 vs. 2–3 vs. ≥ 4) and ISS disease stage during screening (I vs. II vs. III). A median follow-up of 16.9 months revealed that the therapy resulted in the improvement of progression-free survival in the daratumumab plus pomalidomide and dexamethasone group compared with the pomalidomide and dexamethasone group (median 12.4 months [95% CI 8.3–19.3] vs. 6.9 months [5.5–9.3]; hazard ratio 0.63 [95% CI 0·47–0.85], two-sided *p* = 0.0018) [9].

Despite these promising study results, there is only a limited number of publications available in terms of real-world results of pomalidomide treatment. A Japanese group reported on a cohort of 14 RR MM patients who received pomalidomide in combination with low-dose dexamethasone. Unfortunately the pomalidomide therapy was poorly tolerated in this heavily pretreated group, and only 21.4% of the patients were able to continue treatment over 1 year [10]. An Italian working group analyzed the data of 121 MM patients who were administered pomalidomide and low-dose dexamethasone as a median fourth-line therapy. Overall response rate was 43.4%, and median PFS and OS were 8.5 and 14 months, respectively [11]. Maciocia et al retrospectively analyzed the data of 70 patients treated with pomalidomide at five UK centres between 2013 and 2016. 96.5% were refractory to IMiDs, 72.9% were refractory to both an IMiD and bortezomib and 92.9% were refractory to their last line of therapy. The treatment consisted of 28-day cycles of pomalidomide (administered on a daily basis for 1–21) plus dexamethasone (on days 1, 8, 15 and 22), plus or minus a third agent. The overall response rate was measured 52.9%. With a median follow-up of 13.2 months, median progression-free survival was 5.2 months, and median overall survival was 13.7 months. No significant difference was observed in response, survival or tolerability by renal function, age or cytogenetic risk [12]. Charlinski et al reported on 50 RR multiple myeloma patients from Poland who received either pomalidomide treatment with dexamethasone or pomalidomide treatment combined with dexamethasone and bortezomib. Their investigations showed that the overall response rate was 39.1%. Median progression-free survival (PFS) and overall survival (OS) were measured 10.0 and 14.0 months, respectively. No associations between previous treatment with immunomodulatory drugs, bortezomib or stem cell transplant and PFS or OS could be justified [13]. The Spanish PETHEMA-Gem working group investigated the efficacy of pomalidomide plus cyclophosphamide and dexamethasone combination in the RR setting of multiple myeloma. They analyzed the survival data of 100 patients and found that the median PFS and OS were 7.6 and 12.6 months, respectively, which compares favorably with other triplets in the same setting [14]. In a recently published paper, US-based community oncologists evaluated the efficacy of pomalidomide therapy in second line after lenalidomide-based induction. They analyzed the data of 300 relapsing MM patients, from which 126 received pomalidomide-based and 174 received non-pomalidomide therapy as a second line regimen. In pomalidomide and non-pomalidomide cohorts, disease response was 78.6 and 51.7%, respectively (*p* **<** 0.0001). Multivariate adjusted odds of response were 4.5-times greater for the cohort treated with pomalidomide (*p* **<** 0.0001). Median progression-free survival was not reached in the pomalidomide cohort, while it was measured 16.7 months in the non-pomalidomide cohort (log-rank *p* **<** 0.01) [15].

Our Hungarian Myeloma Working Group is highly committed to make research among patients receiving innovative therapies. We recently published our results about the efficacy of lenalidomide, ixazomib, daratumumab and venetoclax-based treatments [16,17,18,19]. As MM patients are usually administered several lines of therapies, there are overlaps between the patient populations of these publications. In this study, we reported eighty-six RR multiple myeloma patients received pomalidomide therapy outside any clinical trials until December 2021. Our treatment protocols were strongly influenced by funding rules of the national health insurance, since pomalidomide was reimbursed only for patients who received over 3 prior lines of treatment and individual requests had to be submitted in each case. As a result, most of our patients were heavily pretreated and received pomalidomide in combination with dexamethasone plus/minus an alkylating agent. Interestingly, the treatment responses and survival results were superior to the findings of the MM03 study and were rather comparable to the data of the OPTIMISMM trial and the Polish Myeloma Group [7,13]. However, there was a trend of superior PFS in our PI combination group. Based on the results of the analysis of data collected from the subjects in each subgroup, we identified R-ISS and extramedullary manifestation of the disease as the most important predictive factors of survival. Patients with adverse genetic alterations showed markedly inferior survival in comparison with those having standard FISH results. Notably, our findings demonstrate that no significant associations can be justified between the number of prior treatment lines or lenalidomide refractoriness and PFS results. Furthermore, patients with impaired renal function showed similar PFS results to those with normal GFR values. Since this population was excluded from most of the clinical trials including OPTIMISMM, ICARIA and APOLLO, our research is the first to give evidence that administration of pomalidomide to MM patients with moderate or severe renal failure is safe, effective and well-tolerable.

Serious adverse events including patients’ deaths occurred in 8 cases (9.31%) which rate is comparable to those observed in the official clinical trials. The predisposing factors of severe infections could be both neutropenia and humoral immunodeficiency due to immunoparesis in MM parients. Fortunately, most of our patients developed no or only minor side effects.

## Data Availability

The raw data supporting the conclusion of this article will be made available by the authors, without undue reservation.
